# A Proposed Systematic Approach to the Cognitive Autopsy for Internists

**DOI:** 10.7759/cureus.20965

**Published:** 2022-01-05

**Authors:** Kathryn E Berlin, Kathlyn E Fletcher

**Affiliations:** 1 Neonatology, Medical College of Wisconsin, Milwaukee, USA; 2 Internal Medicine, Medical College of Wisconsin, Milwaukee, USA; 3 Internal Medicine, Clement J. Zablocki Veterans Affairs Medical Center, Milwaukee, USA

**Keywords:** cognitive error, error, cognition, diagnostic error, general internal medicine

## Abstract

The cognitive autopsy has been a proposed tool for physicians to evaluate misdiagnosis. However, prior iterations of this tool are cumbersome, not designed for the internist, and may cause users to isolate cognition from systems issues. A 10-point tool was created to be utilized individually or by a group when evaluating an adverse event. This could be used with Croskerry’s 2020 “cognitive autopsy” or as a standalone tool for internists. We trialed this tool in large group formats and with individual residents; all reported an improved appreciation of the factors leading to poor outcomes and medical errors.

## Introduction

An autopsy is defined as “an examination of a body after death to determine the cause of death or the character and extent of changes produced by disease” [1]. The process is systematic and includes a thorough examination of external and internal structures to determine what could be improved.

The modern fields of patient safety and quality improvement require a level of evaluation to determine what went wrong so that it can be prevented from happening again. The patient safety movement started with “To Err is Human” in 1999 [2]. Since then, physicians, along with the healthcare system as a whole, have become adept at identifying systems issues that result in near misses or adverse patient events. A whole field has sprung up to identify, avoid, and mitigate errors at the system’s level. Fishbones, Pareto charts, spaghetti plots, and the like have been developed to help us determine where system vulnerabilities exist. 

This systems review is essential to work and not to be underscored. However, it is only one piece of the puzzle. A diagnostic error has not received the same attention and care as systemic errors. A 2015 report from the National Academy of Medicine described this lack of focus on diagnostic errors as a blind spot in our field [3]. One reason for this blind spot may be that diagnostic error is taken and attributed personally. Despite the advances in patient safety, diagnostic error is still often seen as a personal flaw rather than an unavoidable occurrence. There are limited tools to undertake a systematic and depersonalized approach to evaluating these types of errors. 

In 2000, Croskerry, Wears, and Binder outlined a curriculum designed to prevent errors in the Emergency Department [4]. In this work, the team emphasized the importance of developing initiatives that allowed an individual to respond to an error, including identifying practice patterns that indicated error was fast approaching. 

Croskerry would later describe a “cognitive and affective autopsy” in 2005, which was further outlined in a 2020 book [5]. This cognitive autopsy was developed to be a retrospective analytical process that took the place of the maladaptive “guilt, soul-searching, self-recrimination...and despair” that was otherwise associated with diagnostic errors [6]. The goal was to learn from the error and mitigate the second victim syndrome that often develops after serious errors.

Croskerry’s proposed methodology is mainly practical and has been adopted by many institutions, including the New South Wales government. However, it has some limitations: It is not easy to organize thoughts within this schema, which was created with the emergency department in mind. In internal medicine, diagnostic errors may span days to weeks, with multiple players involved requiring a more organized format; The affective and cognitive dispositions outlined in this methodology do not break down the thought process involved with diagnostic cognition as systematically as possible. It can be cumbersome and downright difficult to determine which dispositions were involved in misdiagnosis on initial use; Because of the multifactorial nature of medical errors, it is essential to have a systematic evaluation method.

Croskerry found that, on average, 4 to 6 independent factors are found with each cognitive autopsy [5]. It is essential to have a systematic method to allow for complete discovery. Finally, other internal medicine programs have adapted the cognitive autopsy but isolated the cognition analysis from the systems analysis. This may result in an incomplete understanding of the picture [7].

## Technical report

**Figure 1:**
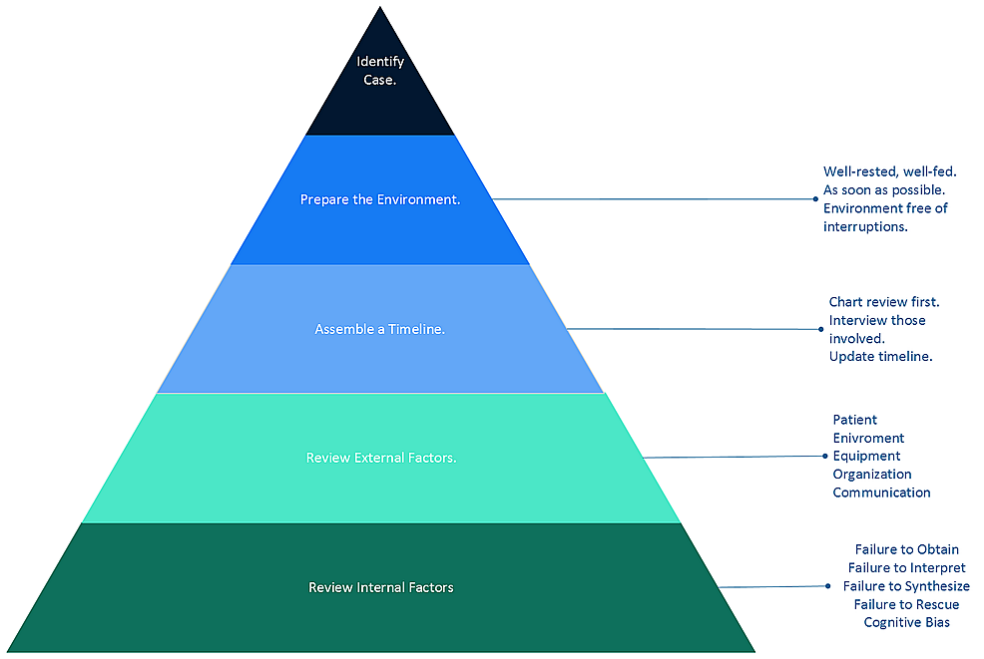
The Cognitive Autopsy

The goal was to create a cognitive autopsy tool that is systematic yet manageable and would allow physicians, including trainees, to perform a retrospective analysis of diagnostic errors in a beneficial manner. Additionally, this tool needed to encompass both systems issues and cognitive errors and could be utilized by individuals or groups during an educational conference such as Morbidity & Mortality. The proposed tool is detailed below and in figure 1.

The cognitive autopsy outlined in this intervention should be initiated similarly to Croskerry proposed. This includes conducting the cognitive autopsy as soon as possible after an event, although it is essential to ensure that the individual feels emotionally prepared to dissect the error. This may require allowing some time to pass. The individual running the cognitive autopsy should be well-rested, well-fed, and in a secluded location, free from interruptions [5]. The case timeline should be reviewed with documentation of all memories, associated feelings and thoughts, and relevant labs, vitals, and imaging. If a moderator assembles a case to be presented to a group, all relevant parties should be interviewed separately. Ultimately, a timeline with relevant information (including vitals, labs, and consults) should be assembled. 

Next, this timeline should be dissected utilizing the 10 domains of a cognitive autopsy (Table 1), including evaluation of both external and internal factors. 

**Table 1:** Two Domains of the Cognitive Autopsy

External Factors (Systems)	Internal Factors (Cognitive)
Patient Factors	Failure to Obtain
Environmental Factors	Failure to Interpret
Equipment Factors	Failure to Synthesize
Organizational Factors	Failure to Rescue
Communication Factors	Cognitive Bias

When initiating the process, participants should start with the external factors. The assembled timeline should be reviewed, looking for any of the following factors that played into the adverse event or near-miss. Patient factors encompass any component of that patient that contributed to an event. This includes patient personality, family, past medical history, medications, etc. An example includes a patient refusing lab draws in the mornings. Environmental factors include temporal factors as well as a physical location. For example, a case that occurred overnight in July or a patient who was located a long walk from the call room would qualify as environmental factors. Equipment factors include both medical equipment and technology. This could be an order that was difficult to locate within the computerized order entry system. Organizational factors include policy & procedures of an institution and cultural factors. Finally, communication factors are any communication (written or oral) that was suboptimal and contributed to an event. Examples include a nurse not paging about an episode of hypotension or a sign-out that did not mention a neutropenic patient. 

After evaluating external factors, the participants should examine those internal factors contributing to the outcome. Failure to obtain refers to a failure to gather any needed information. Was there a question omitted from the history? Was there an organ system that was not examined? Were there labs or imaging that should have been ordered? Failure to interpret referred to an error when the correct information was gathered but not interpreted correctly. For instance, was a piece of history or physical exam glossed over or attributed to a different etiology? Failure to synthesize means that all the correct pieces were gathered and interpreted but that the diagnosis as a whole was not put together correctly. In these cases, one should ask if the wrong illness script was created from the available information or if several diagnoses were considered rather than the correct unifying diagnosis. Failure to rescue should be considered if a decompensation or medical emergency was not promptly identified and intervened upon. For instance, were medications or other interventions that should have been initiated either not done or delayed? Were the wrong medications started? Finally, one should consider any present cognitive biases. Was there evidence of anchoring, premature closure, or other cognitive biases present? 

It is important to evaluate cognitive biases closely because they often play into the previously discussed internal factors. Croskerry discussed that cognitive biases often occur distal to gathering and processing information failures [8]. If cognitive biases are not duly considered, one may latch onto an error in the history or a missed interpretation of a lab result rather than assessing and diagnosing the underlying cognitive bias. This is essential because an appropriate solution requires addressing failure to obtain, interpret, synthesize, or rescue and the underlying cognitive biases.

## Discussion

Our proposed cognitive autopsy has been utilized several times in our internal medicine residency since its inception. Initially, it was introduced during two monthly Morbidity and Mortality conferences. A moderator (one of the authors) assembled a case to be presented to a group. The moderator performed a chart review and assembled a timeline. She then interviewed all relevant parties separately. Finally, the timeline was presented to a group of physicians and medical students. After reviewing the case, the group worked through each step of the cognitive autopsy. Subjectively, many residents reported a greater understanding of the complex dynamics at play in medical errors and appreciated the analytic approach to cognitive errors. Residents have also utilized this cognitive autopsy tool individually when reviewing cases where they made errors or had “bad outcomes”. In these instances, the resident performing the cognitive autopsy was initially encouraged to document all memories, associated feelings, and thoughts prior to performing a chart review. These residents would then individually dissect their thought process and the environment contributing to the outcome using Table 1. Residents who utilized this method appreciated the tool’s ease and noted they planned to continue using it for future cases. 

We believe this cognitive autopsy is a systematic and easy-to-use method to evaluate diagnostic errors made by the internist and hope that it may be a valuable learning opportunity for other physicians. It would be beneficial to perform a formal study to validate this tool. Additionally, we hope to incorporate our cognitive autopsy into future Morbidity & Mortality Conferences and individual resident assessments.

## Conclusions

We believe this tool is a systematic and easy-to-use method to evaluate diagnostic errors made by the internist and hope that it may be a valuable learning opportunity for other physicians.
